# Molecular transmission network and spatial analysis of HIV/AIDS in Hainan, China: a population-based study

**DOI:** 10.3389/fmicb.2026.1907224

**Published:** 2026-08-20

**Authors:** Zhenjiao Zhang, Xin Chen, Huayue Liang, Ruolan Fu, Ming Chen, Baoshan Ke, Qiongjun Xu, Jindong Ding Petersen, Zhaoqian Wang, Dee Yu

**Affiliations:** 1 Key Laboratory of Tropical Translational Medicine of Ministry of Education, School of Public Health, Hainan Academy of Medical Sciences, Hainan Medical University, Haikou, Hainan, China; 2 The First People’s Hospital of Qinzhou, Qinzhou, Guangxi, China; 3 HIV/AIDS Prevention and Control Unit, Haikou Center for Disease Control and Prevention, Haikou, Hainan, China; 4 Red Cross Society of Haikou City, Haikou, Hainan, China; 5 Hainan Provincial Center for Disease Control and Prevention, Haikou, Hainan, China

**Keywords:** cross-regional transmission, Hainan, HIV-1, molecular transmission network, spatial analysis

## Abstract

**Background:**

The geographical distribution of HIV in Hainan Province is highly heterogeneous. Yet the patterns of cross-regional transmission remain unclear. To address this issue, we integrated HIV transmission network analysis with spatial analysis to characterize cross-regional transmission patterns across the province.

**Methods:**

We analyzed 2,135 HIV-1 *pol* gene sequences obtained from HIV/AIDS patients reported in Hainan Province between 2021 and 2025. A molecular transmission network was constructed using a genetic distance threshold of 1.5% to identify cross-regional links among network nodes. Spatial autocorrelation analysis was subsequently performed to characterize the transmission patterns within and across cities in Hainan Province.

**Results:**

Among 2,135 HIV-1 *pol* sequences, 1,396 (65.39%) sequences were included in the molecular transmission network. Older aged (≥50 years), no drug resistance, and infection with CRF07_BC and CRF65_cpx were independently factors associated with a higher likelihood of clustering. Global spatial autocorrelation analysis indicated significant spatial dependence in clustering rates across Hainan (Moran’s *I* = 0.256, *P* < 0.05). Local analysis identified Wuzhishan as a high-low cluster for all subtypes, and Danzhou specifically for CRF07_BC. Network linkage analysis revealed intensive intracity transmission within Haikou and Sanya across all subtypes, with the strongest intercity links between these two cities for CRF07_BC, CRF01_AE, and CRF65_cpx. Of the 1,396 nodes, 79.87% displayed intercity linkage, and female sex was less likely linked, while infection with CRF65_cpx increased linkage likelihood.

**Conclusion:**

This study characterizes the patterns of cross-regional HIV transmission in Hainan Province, highlighting the Haikou-Sanya link as a crucial intervention point. Enhanced regional surveillance and coordinated prevention strategies are recommended for this transmission route.

## Introduction

The HIV epidemic remains a major global public health challenge. According to the Joint United Nations Programme on HIV/AIDS (UNAIDS), 40.8 million people worldwide were living with HIV in 2024, and 1.3 million new HIV infections occurred in the same year. In China, approximately 1.36 million people living with HIV by the end of 2024, with 65,000 newly reported cases. The persistent burden of HIV epidemic continues to impede progress toward the goal of ending the AIDS epidemic by 2030 (De Lay et al., 2021). In recent years, China’s efforts to prevent and control AIDS have yielded significant progress (General Office of the State Council, 2024). However, the spatial distribution of HIV/AIDS has gradually shifted from a random pattern to a clustered one, with increasing spatial dependence across regions (Zulu et al., 2014).

Studies in China have shown that the HIV/AIDS epidemic exhibits significant spatial heterogeneity, shaped by both geographical factors and population behaviors (Wu et al., 2025). Furthermore, multiple studies have indicated that the HIV/AIDS epidemic in China not only involves cross-regional transmission spanning multiple provinces but also exhibits significant cross-border transmission characteristics in border areas (Yin et al., 2021; Li et al., 2022). Accordingly, geographically targeted interventions have become an important strategy for HIV transmission prevention and control (Wirtz et al., 2017). Compared with universal interventions, such approaches may be more cost-effective in reducing HIV transmission (Anderson et al., 2014). Therefore, further geographic analyses are crucial for identifying dynamic transmission hotspots and informing region-specific interventions region-specific intervention strategies.

Molecular transmission network analysis has become an important tool for HIV prevention and control (Brenner et al., 2013). By assessing the genetic similarity of viral gene sequences, HIV molecular transmission network can be constructed to identify potential transmission chains and characterize transmission patterns at the molecular level (Hassan et al., 2017). Compared with traditional epidemiological methods, molecular transmission networks can uncover hidden transmission patterns that may otherwise remain undetected. When integrated with sociodemographic and behavioral data, this approach can further help identify hidden high-risk populations, thereby providing a scientific basis for targeted interventions (Arantes et al., 2021).

HIV molecular transmission network analysis has been widely applied across countries and populations. For example, a study in Botswana found that approximately 68% of transmission lineages spanned multiple communities, revealing a highly diverse network structure and widespread cross-community transmission (Novitsky et al., 2020). In China, transmission network analysis has been shown to identify geographic clusters of HIV/AIDS cases and key HIV/AIDS individuals at high risk of transmission (Yang et al., 2025). Studies of spatially resolved HIV molecular transmission networks have provided further evidence for the spatial heterogeneity of HIV transmission (Yuan et al., 2022). Therefore, integrating molecular transmission networks with spatial analysis can help identify high-risk areas, optimize resource allocation, and improve the cost-effectiveness of intervention strategies.

The HIV epidemic in Hainan Province exhibits notable geographical heterogeneity. Yet, the patterns of cross-regional transmission remain poorly understood. With the establishment of the Hainan Free Trade Port, increasing inter-provincial and cross-border population mobility, together with the province’s rapidly growing tourism industry, may heighten the risk of infectious disease transmission, including HIV. Population mobility, rapid economic development, and the expansion of transportation networks are important drivers shaping the dynamics of HIV transmission (Kate Grabowski et al., 2020).

Current HIV-related research, both in China and internationally, has largely focused on behaviors, attitudes, and factors associated with HIV infection (Sulistina et al., 2024). However, previous HIV spatial analyses in China have largely relied on case data from selected years. They have also primarily focused on the southwestern region. Comprehensive provincial level spatial assessments remain limited. In Hainan Province, relevant evidence has remained limited over the past decade. To address this gap, we integrated molecular transmission networks analysis, epidemiological data, and spatial information to comprehensively characterize the transmission patterns and spatial distribution of the HIV-1 epidemic in Hainan Province. Our findings may provide a scientific basis for developing geographically targeted prevention and control strategies.

## Materials and methods

### Study setting and participants

This study employed a cross-sectional design, and recruited individuals from 18 cities and counties across Hainan Province between January 2021 and May 2025 using respondent-driven sampling (RDS).

Participants were eligible if they met the following criteria: (1) aged 18 years or older at the time of the survey; (2) diagnosed with HIV-1 infection; and (3) provided written informed consent form. Individuals were excluded if they were unwilling to complete the epidemiological investigation or provide a blood sample.

**FIGURE 1:**
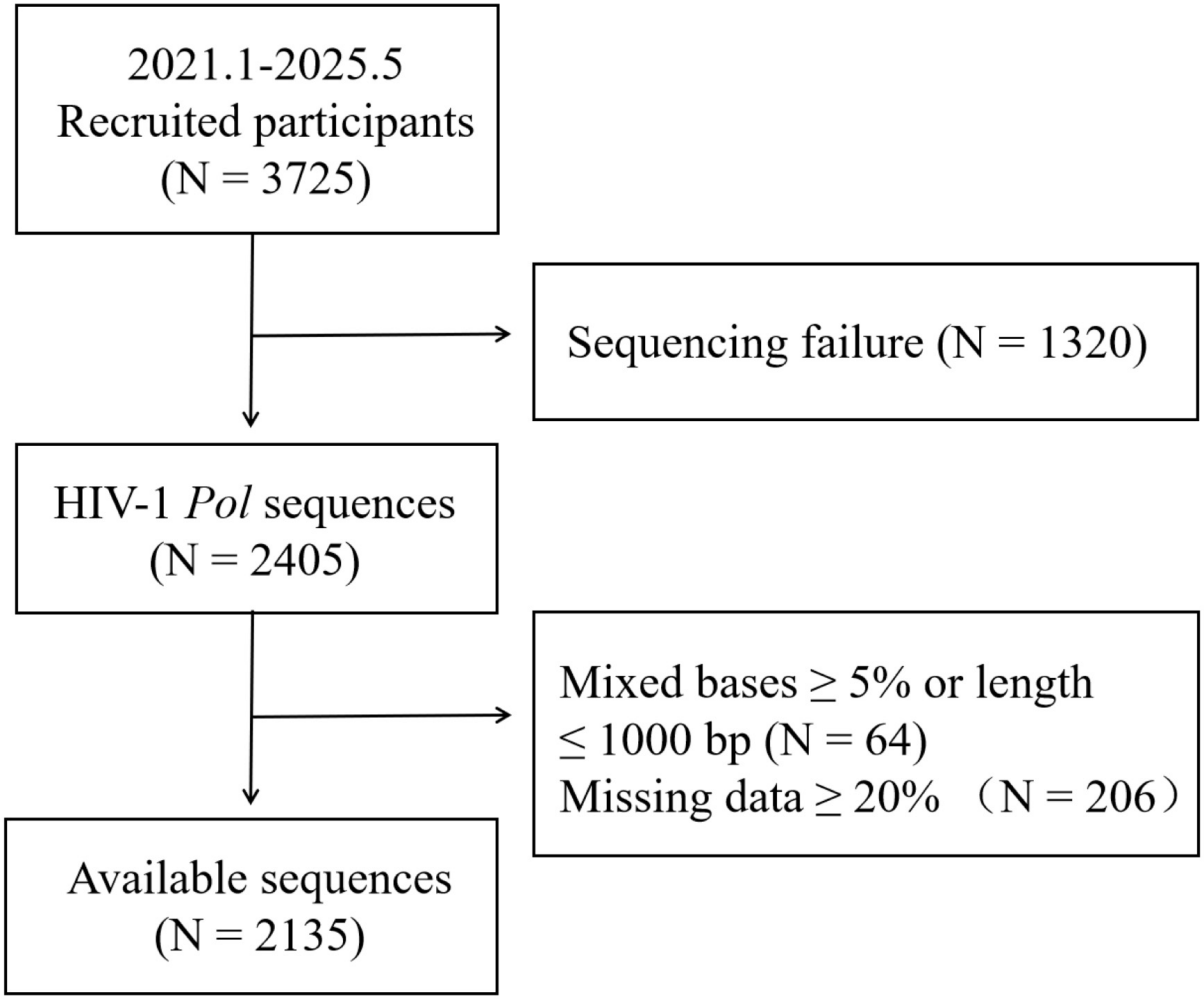
Study enrollment flow diagram.

Among 3,725 people living with HIV, HIV-1 *pol* sequences were successfully obtained from 2,405 samples, accounting for 64.6% of the total. After excluding 64 sequences that failed quality control criteria (with mixed bases ≥5% or sequence length ≤1,000 bp), and 206 cases with missing data ≥20%, 2,135 cases were ultimately included in the final analysis (Figure 1).

### Data sources

Demographic information on the study participants was downloaded from the Basic Information System for HIV/AIDS Prevention and Control, including sex, ethnicity, age, education, occupation, transmission routes, marital status, baseline CD4^+^ T-cell count, viral load, and other relevant information. These demographic, clinical, and virological variables were selected based on their potential relevance to HIV molecular transmission dynamics and have been widely evaluated in previous HIV molecular network studies (Poon, 2016; Günthard et al., 2025).

### Sample collection, nucleic acid extraction, and PCR amplification

Peripheral venous blood samples were collected from study participants between January 2021 and May 2025. After plasma was isolated, the samples were stored at −80 °C. Nucleic acids were extracted using a fully automated nucleic acid extractor and corresponding reagent kits (TIANLONG, Xi’an, China). The complete protease and reverse transcriptase region of the HIV-1 *pol* gene (position 2086 to 3441 in reference strain HXB2, 1356 bp in length) was amplified by reverse transcription-polymerase chain reaction (RT-PCR) using primer sets provided by Sangon Biotech. The amplified products were then sent to Sangon Bioengineering Co., Ltd., for sequencing (Song et al., 2018).

### Gene sequence processing and subtype determination

The *pol* gene fragments were assembled using sequencher v.5.4.6 and subsequently uploaded to the HIV Databases (HIV Databases^1^) to align. The aligned sequences were imported into BioEdit software, and trimmed based on the HXB2 reference strain. Preliminary subtyping was performed using the online HIV subtyping tool COMET^2^ and the HIV China Genomic Sequence Database^3^. Sequences that yielded discordant preliminary subtyping results were further analyzed using maximum likelihood (ML) phylogenetic trees to confirm subtype. The reference dataset contained at least three full-length sequences for each HIV-1 subtype (A, B, C, D, F, G, H, J, K), as well as other sequences representing any additional subtypes identified during preliminary subtyping. Three group N sequences served as outgroups. Sequences that formed a cluster with reference sequences (SH-like support > 0.9) were classified as the same subtype. Sequences that could not be classified into specific subtypes were designated as unique recombinant forms (URFs) (Price et al., 2010). Finally, the ML tree was visualized using FigTree V1.4.4.

### HIV-1 molecular transmission network analysis

In this study, HIV-TRACE was used to calculate the genetic distance of sequences. According to the Technical Guideline for HIV Transmission Network Monitoring and Intervention released by the Chinese Center for Disease Control and Prevention (Centers for Disease Control and Prevention, 2025), a genetic distance threshold of 1.5% was selected to determine all possible infected individuals in the transmission network, which could infer transmission events that occurred within 5–15 years. The transmission network was generated using Cytoscape (v3.6.1) software (Li et al., 2021).

### Statistical analysis

Multiple imputation for missing data was performed using R software (version 4.5.0). Categorical variables were described using frequencies and percentages, and logistic regression analysis was employed to identify factors associated with clustering. Variables with *P* < 0.05 in the univariate logistic regression analysis were included in the multivariate logistic regression analysis. All statistical significance test results were expressed as *P*-values, and *P* < 0.05 (two-tailed) was considered statistically significant.

### Spatial analysis

Global and local spatial autocorrelation analyses were conducted using GeoDa 1.16 (Anselin et al., 2006). The Global Moran’s *I* index was employed to assess the spatial clustering of the five main HIV-1 subtypes at the city level. The city variable was defined as the city where the HIV-1 sample was collected, representing the current residence of participants. The distribution of HIV-1 subtype was visualized using pie charts. Individuals were classified as having intercity transmission if they had at least one molecular link with an individual from a different sampling city within the molecular transmission network. Intercity transmission within molecular networks were visualized using a transmission matrix heatmap and a flow map. An additional sample-size adjusted analysis was performed to assess the potential impact of unequal sampling sizes among cities (Borgatti and Everett, 1997). The normalized linkage intensity was calculated as:


$${\text{LI}}_{\text{ij}}=\frac{{\text{E}}_{\text{ij}}}{\sqrt{{\text{N}}_{\text{i}}\times {\text{N}}_{\text{j}}}}$$

Where E_*ij*_ is the number of observed molecular edges between sequences from city i and city j. N_*i*_ and N_*j*_ are the effective number of sequences with at least one molecular link (degree ≥ 1) in city i and city j, respectively.

The Global Moran’s *I* index reflects the similarity of attribute values among spatially adjacent units. A Moran’s *I* > 0 with *Z* > 1.96 indicates a clustered distribution; Moran’s *I* < 0 with *Z* < −1.96 indicates a dispersed distribution; and Moran’s *I* = 0 with Z between −1.96 and 1.96 indicates a random distribution with no spatial autocorrelation. Local indicators of spatial association (LISA) cluster maps were used to visualize the spatial clustering results. Four types of spatial clusters were identified: high-high, low-low, high-low, and low-high. high-high and low-low clustering indicate that the clustering rate in a given city is similar to that of its surrounding areas (i.e., both high or both low). High-low and low-high clustering indicate that the clustering rate in a given city is higher (or lower) than that of its surrounding areas. Non-significant areas indicate that clustering rates are randomly distributed across regions.

## Results

### Demographic characteristics of the participants

Among the 2,135 participants, 89.70% were male, 43.42% were diagnosed at age ≤29 years, 41.83% had a university education or above, 60.14% were casual workers, and 62.72% were unmarried (Table 1).

**TABLE 1:** Demographic and clinical factors associated with clustering within the HIV-1 transmission network.

Variables	Total (*n* = 2135, %)	Clustering (*n* = 1396, %)	Univariate analysis		Multivariate analysis	
			*P*	OR (95% CI)	*P*	aOR (95% CI)
Sex						
Male	1915 (89.70)	1267 (66.16)	Ref.		Ref.	
Female	220 (10.30)	129 (58.64)	0.027	0.725 (0.545–0.964)	0.153	0.791 (0.573–1.091)
Diagnosis age (year)						
≤29	927 (43.42)	590 (63.65)	Ref.		Ref.	
30–39	521 (24.40)	345 (66.22)	0.326	1.120 (0.894–1.403)	0.176	1.180 (0.928–1.502)
40–49	291 (13.63)	173 (59.45)	0.197	0.837 (0.640–1.097)	0.503	0.906 (0.677–1.211)
≥50	396 (18.55)	288 (72.73)	0.001	1.523 (1.176–1.972)	<0.001	1.785 (1.317–2.419)
Ethnicity						
Han	1863 (87.26)	1233 (66.18)	Ref.		Ref.	
Others	272 (12.74)	163 (59.93)	0.043	0.764 (0.589–0.992)	0.074	0.779 (0.593–1.024)
Education level						
Junior school and below	761 (35.64)	496 (65.18)	Ref.			
High school	481(22.53)	325 (67.57)	0.386	1.113 (0.874–1.418)		
University and above	893 (41.83)	575 (64.39)	0.738	0.966 (0.789–1.183)		
Occupation						
Regular worker	851 (39.86)	544 (63.92)	Ref.			
Casual worker	1284 (60.14)	852 (66.36)	0.248	1.113 (0.928–1.335)		
Marital status						
Unmarried	1339 (62.72)	867 (64.75)	Ref.			
Married	556 (26.04)	382 (68.71)	0.098	1.195 (0.967–1.477)		
Divorced/widowed	240 (11.24)	147 (61.25)	0.298	0.861 (0.649–1.142)		
Infection routes						
Heterosexual contact	903 (42.30)	575 (63.68)	Ref.			
Homosexual contact	1170 (54.80)	781 (66.75)	0.144	1.145 (0.955–1.374)		
Intravenous drug use	48 (2.25)	31 (64.58)	0.899	1.040 (0.567–1.908)		
Others	14 (0.66)	9 (64.29)	0.962	1.027 (0.34–3.090)		
Cross-regional migrants						
No	1280 (60.00)	856 (66.88)	Ref.			
Yes	855 (40.00)	530(61.99)	0.077	0.849 (0.708–1.018)		
Duration of treatment since initial ART (years)						
<1	845 (39.58)	574 (67.93)	Ref.			
1–3	570 (26.70)	363 (63.68)	0.098	0.828 (0.662–1.035)		
>3	720 (33.72)	459 (63.75)	0.082	0.830 (0.673–1.024)		
Baseline CD4+ T counts (cells/μL)						
<200	681 (31.90)	420 (61.67)	Ref.		Ref.	
200–349	766 (35.88)	509 (66.45)	0.059	1.231 (0.992–1.526)	0.181	1.169 (0.930–1.470)
≥350	688 (32.22)	467 (67.88)	0.016	1.313 (1.051–1.640)	0.050	1.271 (1.000–1.615)
Sampling viral load (copies/ml)						
<50	1373 (64.31)	879 (64.02)	Ref.		Ref.	
50–999	129 (6.04)	82 (63.57)	0.918	0.981 (0.674–1.427)	0.828	1.045 (0.703–1.553)
≥1000	633 (29.65)	435 (68.72)	0.040	1.235 (1.010–1.509)	0.167	1.161 (0.939–1.435)
Number of non-marital sex partners						
0	1303 (61.03)	866 (66.46)	Ref.			
1–4	688 (32.22)	435 (63.23)	0.149	0.868 (0.715–1.052)		
≥5	144 (6.74)	95 (65.97)	0.906	0.978 (0.680–1.407)		
Number of homosexual partners						
0	973 (45.57)	618 (63.51)	Ref.		Ref.	
1–4	509 (23.84)	320 (62.87)	0.806	0.973 (0.779–1.215)	0.615	0.935 (0.718–1.217)
≥5	653 (30.59)	458 (70.14)	0.006	1.349 (1.091–1.668)	0.108	1.231 (0.955–1.585)
Infection of other STD						
No	1615 (75.64)	1069 (66.19)	Ref.			
Yes	404 (18.92)	259 (64.11)	0.430	0.912 (0.726–1.146)		
Unknown	116 (5.43)	68 (58.62)	0.098	0.724 (0.493–1.062)		
Drug resistance						
No	1713 (80.23)	1171 (68.36)	Ref.		Ref.	
Yes	422 (19.77)	225 (53.32)	<0.001	0.529 (0.426–0.657)	<0.001	0.377 (0.294–0.482)
HIV-1 subtypes						
CRF01_AE	880 (41.22)	527 (59.89)	Ref.		Ref.	
CRF07_BC	764 (35.78)	539 (70.55)	<0.001	1.605 (1.306–1.971)	<0.001	1.528 (1.229–1.900)
CRF08_BC	95 (4.45)	62 (65.26)	0.309	1.258 (0.808–1.961)	0.881	1.036 (0.649–1.655)
CRF55_01B	103 (4.82)	72 (69.90)	0.050	1.556 (1.000–2.421)	0.018	1.744 (1.101–2.763)
CRF65_cpx	99 (4.64)	87 (87.88)	<0.001	4.856 (2.617–9.013)	<0.001	10.392 (5.386–20.053)
Others	194 (9.09)	109 (56.19)	0.343	0.859 (0.627–1.176)	0.366	0.862 (0.624–1.190)

ART, antiretroviral therapy; STD, sexually transmitted diseases; OR, odds ratio; aOR, adjusted odds ratio; CI, confidence interval; Ref., reference group; CRF, circulating recombinant form.

Regarding HIV transmission routes, heterosexual contact and homosexual contact accounted for 42.30% and 54.80%, respectively. ART was initiated within 1 year of diagnosis in 39.58% of participants, and 18.92% had other sexually transmitted disease (STD). In terms of clinical characteristics, 64.31% had a viral load below 50 copies/mL, and 19.77% developed drug resistance. In this study, a total of 30 subtypes were identified, with the most prevalent being CRF01_AE (41.22%, *n* = 880), followed by CRF07_BC (35.78%, *n* = 764) (Table 1).

### HIV-1 genetic transmission networks

**FIGURE 2:**
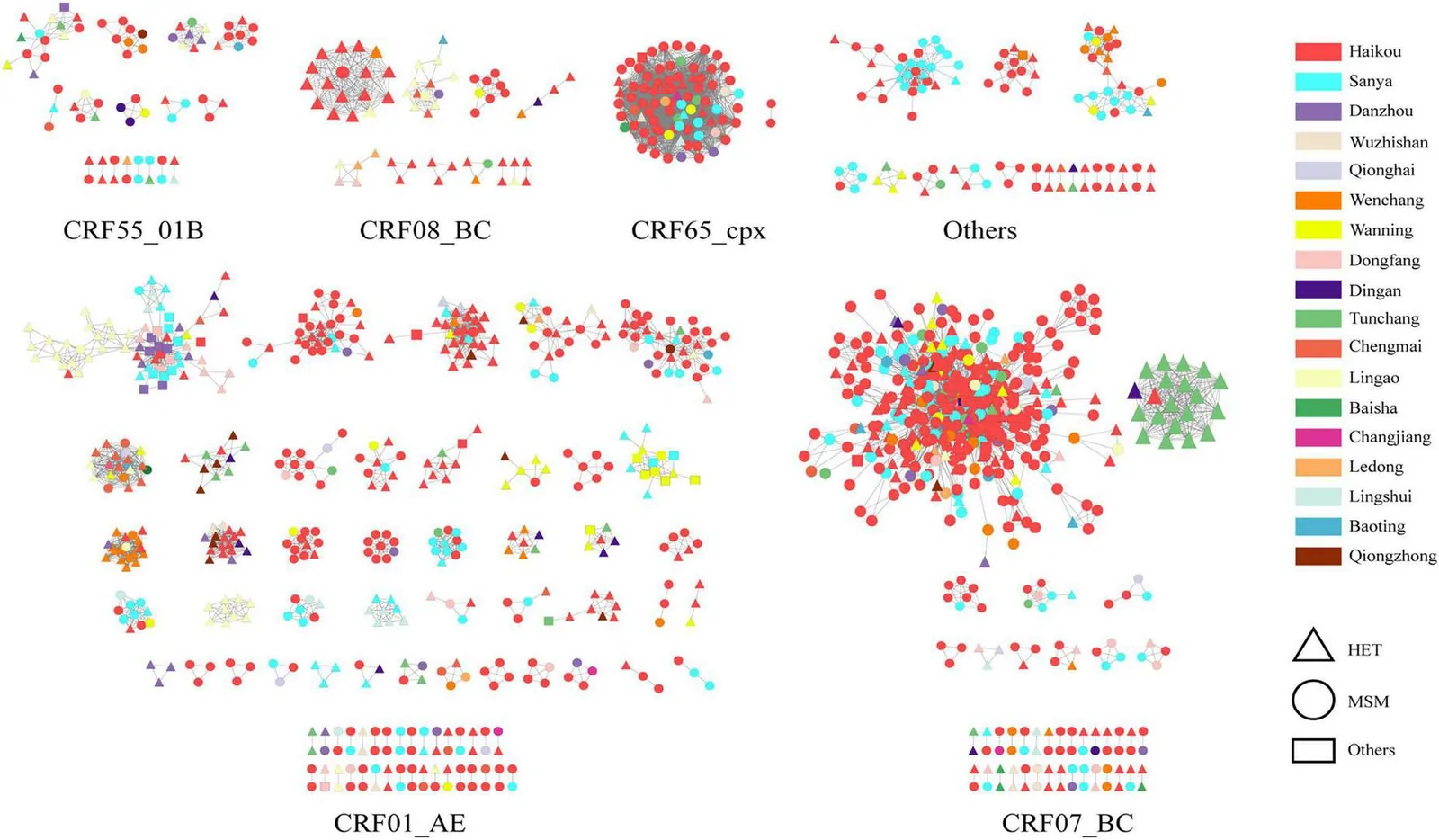
Molecular transmission networks of the HIV-1 subtypes. MSM, men who have sex with men; HET, heterosexual transmission; Others, other transmission routes (including intravenous drug use, mother-to-child transmission, blood transmission, and unknown transmission routes).

Of the 2,135 sequences, 1,396 (65.39%) fell into the molecular transmission network, forming 165 clusters comprising 9,527 edges. Among the cases in the network, 162 nodes were linked to only one sequences, whereas 1,234 were linked to ≥2 sequences. CRF01_AE showed the highest clustering rate (46.60%), forming 76 clusters, followed by CRF07_BC (41 clusters, 24.85%), CRF55_01B (17 clusters, 10.30%), CRF08_BC (11 clusters, 6.67%), and CRF65_cpx (2 clusters, 1.21%). The remaining clusters (18 clusters, 10.91%) were attributed to other subtypes (Figure 2).

### Spatial distribution characteristics of HIV-1 subtypes

**FIGURE 3:**
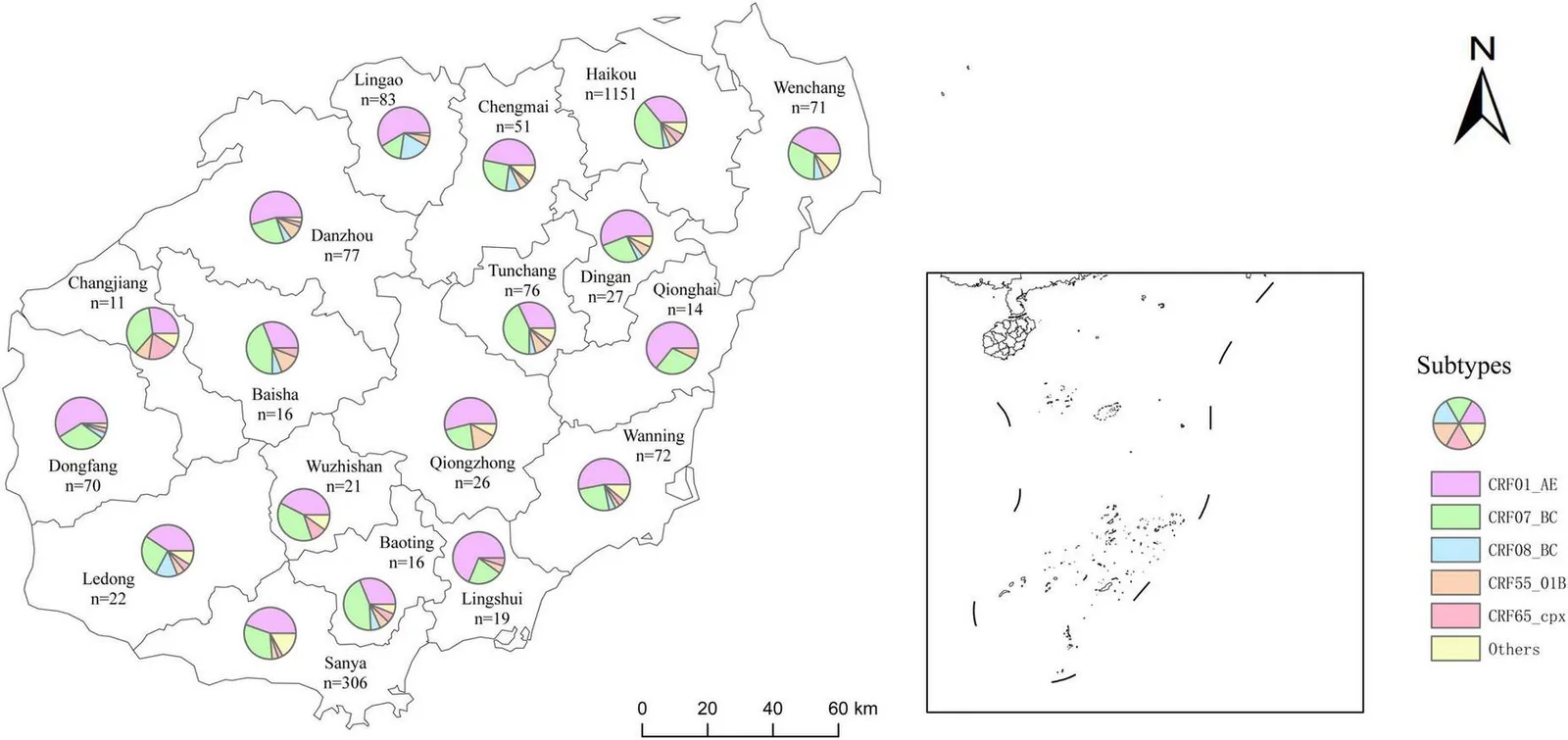
Spatial distribution of HIV-1 subtypes by city and county in Hainan Province, China. Pie charts illustrate the proportions of CRF01_AE, CRF07_BC, CRF08_BC, CRF55_01B, CRF65_cpx, and other HIV-1 subtypes in each county. Map source: National Geospatial Information Public Service Platform of China (https://www.tianditu.gov.cn/), Approval No. GS (2024) 0650.

Among the 2,135 individuals, 53.91% were from Haikou (*n* = 1,151), followed by Sanya (14.33%, *n* = 306), while relatively few cases were reported in the remaining cities. CRF01_AE and CRF07_BC were widely distributed and constituted the predominant subtypes across the majority of prefectures. A limited diversity of subtypes was observed in Qiongzhong, Qionghai, and Wuzhishan. Specifically, CRF08_BC was primarily localized in Lingao, Ledong, and Chengmai. CRF55_01B was prevalent in Qiongzhong, Baisha, and Changjiang. CRF65_cpx was concentrated in Changjiang and Wuzhishan (Figure 3).

### Factors associated with clustering

Multivariable logistic regression showed that age, drug resistance and HIV-1 subtype were independent factors associated with the likelihood of clustering (Table 1). The interaction analysis between HIV-1 subtypes and drug resistance revealed a significant interaction effect (*P* < 0.001), which was particularly pronounced for the CRF65_cpx (OR = 81.062, 95%CI:12.955–685.573) (Supplementary Table 1). Therefore, subtype-specific stratified analyses were conducted (univariable screening results are provided in Supplementary Table 2).

For CRF01_AE, individuals aged ≥50 years at diagnosis had a higher probability of clustering (aOR = 2.193, 95%CI: 1.269–3.815), whereas individuals of other ethnic groups (aOR = 0.580, 95%CI: 0.378–0.889), cross-regional migrants (aOR = 0.642, 95%CI: 0.472–0.872), and occurring drug resistance (aOR = 0.315, 95%CI: 0.214–0.458) showed a lower likelihood of clustering (Supplementary Figure 1A).

For CRF07_BC, individuals with a high school education had a higher risk of clustering (aOR = 1.972, 95%CI: 1.213–3.245). Drug resistance remained correlated with a lower probability of clustering in CRF07_BC (aOR = 0.262, 95%CI: 0.169–0.402) (Supplementary Figure 1B).

For other HIV-1 subtypes, females exhibited a lower risk of clustering (aOR = 0.484, 95%CI: 0.241–0.961) (Supplementary Figure 1C). After excluding the CRF65_cpx from the pooled other subtypes, individuals aged 40–49 years (aOR = 0.464, 95%CI: 0.216–0.990), and those with drug resistance (aOR = 0.571, 95%CI: 0.332–0.985) had a significantly lower likelihood of clustering (Supplementary Figure 1D).

### Spatial patterns of clustering rates

**FIGURE 4:**
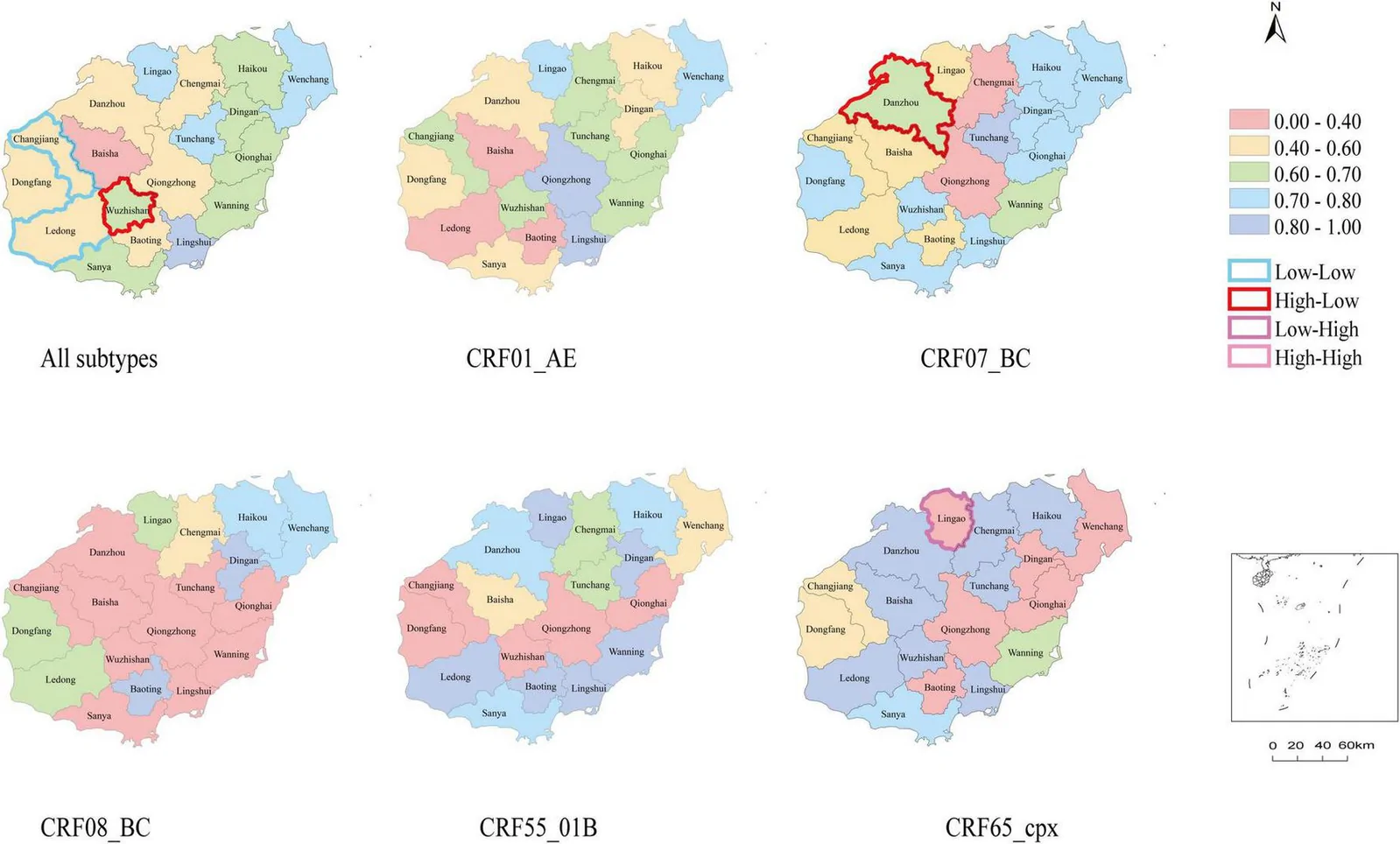
Spatial distribution of HIV-1 subtype clustering rates and their aggregation areas. Lower (higher) values indicate lower (higher) clustering rates. The color-outlined regions represent different types of aggregation areas identified by local spatial autocorrelation analysis. Map source: National Geospatial Information Public Service Platform of China (https://www.tianditu.gov.cn/), Approval No. GS (2024) 0650.

Global spatial autocorrelation analysis demonstrated significant spatial dependence in the clustering rates (Moran’s *I* = 0.256, *P* < 0.05) (Table 2). Clustering rates varied across cities, with higher clustering observed in eastern Hainan for all subtypes, as well as for CRF01_AE and CRF07_BC (Figure 4). High clustering rates of CRF08_BC and CRF55_01B were mainly observed in northern and southern regions, respectively. Local spatial autocorrelation analysis identified low–low cluster for all subtypes in Changjiang and Ledong, a high–low cluster for all subtypes in Wuzhishan, for CRF07_BC in Danzhou, and a low-high cluster in Lingao for CRF65_cpx (Figure 4).

**TABLE 2:** Global spatial autocorrelation of HIV-1 subtypes in Hainan Province.

Subtype	Moran’s *I*	*Z*-value	*P*-value
All subtypes	0.256	2.231	0.026
CRF01_AE	0.073	0.993	0.321
CRF07_BC	−0.163	−0.742	0.458
CRF08_BC	0.061	0.812	0.417
CRF55_01B	−0.021	0.258	0.797
CRF65_cpx	−0.156	−0.654	0.513
Others	0.040	0.674	0.500

### HIV-1 transmission links between and within cities

**FIGURE 5:**
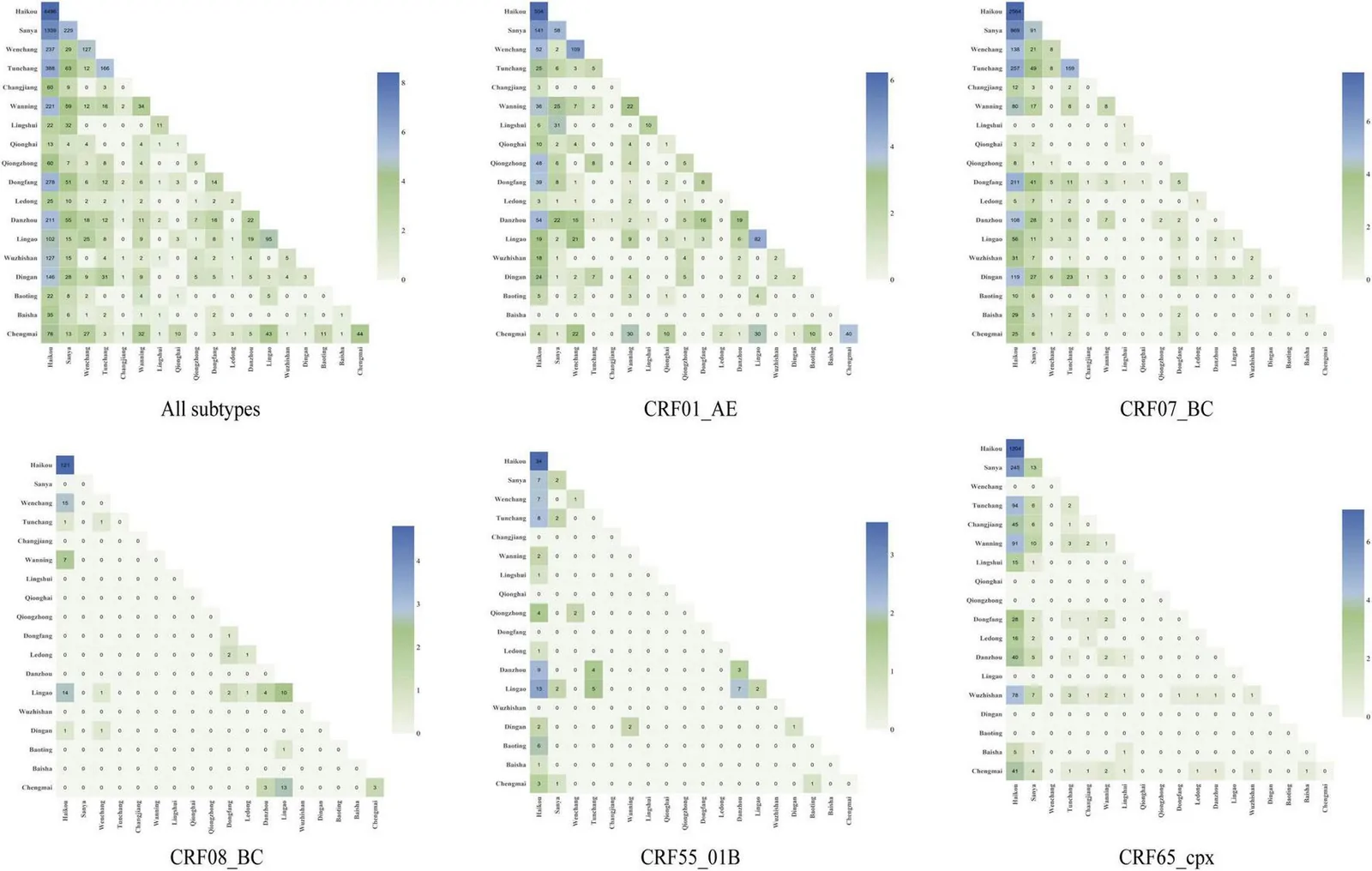
Transmission matrix heatmap for genetic links of HIV-1 among cities and counties in Hainan. Intracity and intercity transmission in the molecular transmission networks of different HIV-1 subtypes are presented using a transmission matrix heatmap, where cell color intensity at the intersections of paired cities and counties represents the number of HIV-1 transmission linkages. A base-10 logarithmic color scale is used to enhance color contrast.

Based on 1,396 sequences within the network, heatmap analysis of the HIV-1 transmission matrix showed that five subtypes exhibited strong intracity transmission in Haikou, while CRF01_AE showed relatively strong intracity transmission in Sanya, Wenchang, Lingao, and Chengmai, and CRF07_BC in Sanya and Tunchang. Within the transmission network, most cities where CRF01_AE and CRF07_BC were predominant formed close transmission links with Haikou, whereas CRF65_cpx was mainly transmitted between Haikou and Sanya, Tunchang, Wanning, and Wuzhishan (Figure 5). After adjusting for city-specific sample sizes, the Haikou-Sanya pair consistently exhibited relatively high molecular linkage intensities among all intercity pairs across the major subtypes, including CRF01_AE, CRF07_BC, and CRF65_cpx (Supplementary Figure 2). The normalized linkage patterns were generally consistent with those observed from the original molecular link counts.

**FIGURE 6:**
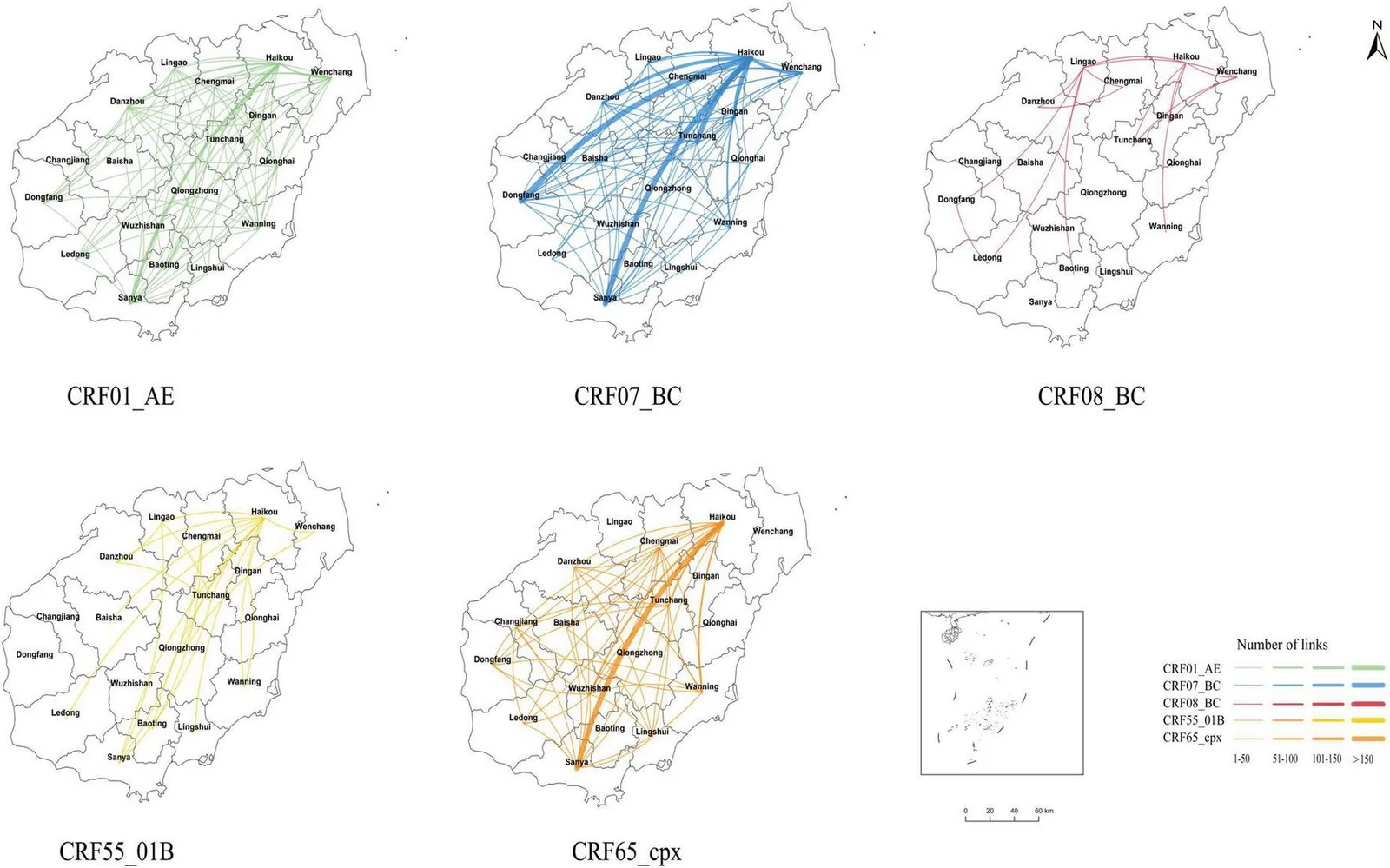
Flow map of HIV transmission linkage strength within the HIV-1 genetic transmission network across cities in Hainan Province. Line thickness denotes the number and strength of intercity connections. Map source: National Geospatial Information Public Service Platform of China (https://www.tianditu.gov.cn/), Approval No. GS (2024) 0650.

The spatial patterns of intercity transmission varied by subtype. The flow map showed that CRF01_AE and CRF07_BC had the widest transmission range, with the strongest linkage Haikou-Sanya. For CRF07_BC, additional strong intercity transmission was observed Tunchang–Haikou and Dongfang–Haikou. CRF55_01B showed stronger transmission mainly in Haikou and adjacent cities, while CRF08_BC showed relatively weaker intercity transmission. For CRF65_cpx, strong intercity transmission was observed Haikou–Sanya (Figure 6).

Of the 1,396 individuals within the network, 1,115 were involved in intercity transmission. Multivariable logistic regression analysis revealed that female sex (aOR = 0.548, 95%CI: 0.363–0.827) was associated with a lower likelihood of intercity transmission. Unknown STD status (aOR = 2.512, 95%CI: 1.125–5.610) and CRF65_cpx infection (aOR = 5.371, 95%CI: 1.793–16.087) were associated with a higher likelihood (Table 3).

**TABLE 3:** Analysis of factors influencing intercity transmission among HIV-1 individuals in the network.

Variables	Total (*n* = 1396)	Cross-city linked (*n* = 1115, %)	Univariate analysis		Multivariate analysis	
			*P*	OR (95% CI)	*P*	aOR (95% CI)
Sex						
Male	1267	1026 (80.98)	Ref.		Ref.	
Female	129	89 (68.99)	0.001	0.523 (0.351–0.779)	0.004	0.548 (0.363–0.827)
Diagnosis age (year)						
≤29	590	471 (79.83)	Ref.			
30–39	345	281 (81.45)	0.547	1.109 (0.791–1.555)		
40–49	173	140 (80.92)	0.751	1.072 (0.698–1.647)		
≥50	288	223 (77.43)	0.412	0.867 (0.616–1.220)		
Ethnicity						
Han	1233	979 (79.40)	Ref.			
Others	163	136 (83.44)	0.228	1.307 (0.846–2.020)		
Education level						
Junior school and below	496	394 (79.44)	Ref.			
High school	325	264 (81.23)	0.528	1.12 (0.787–1.595)		
University and above	575	457 (79.48)	0.986	1.003 (0.745–1.350)		
Occupation						
Regular worker	544	436 (80.15)	Ref.			
Casual worker	852	679 (79.69)	0.837	0.972 (0.743–1.272)		
Marital status						
Unmarried	867	699 (80.62)	Ref.			
Married	382	305 (79.84)	0.749	0.952 (0.704–1.287)		
Divorced/widowed	147	111 (75.51)	0.154	0.741 (0.491–1.119)		
Infection routes						
Heterosexual contact	575	449 (78.09)	Ref.			
Homosexual contact	781	631 (80.79)	0.221	1.180 (0.905–1.540)		
Intravenous drug use	31	28 (90.32)	0.118	2.619 (0.783–8.757)		
Others	9	7 (77.78)	0.982	0.982 (0.202–4.787)		
Cross-regional migrants						
No	856	691 (80.72)	Ref.			
Yes	530	424 (80.00)	0.317	0.873 (0.669–1.139)		
Duration of treatment since initial ART (years)						
<1	574	457 (79.62)	Ref.			
1–3	363	280 (77.13)	0.367	0.864 (0.628–1.187)		
>3	459	378 (82.35)	0.267	1.195 (0.872–1.636)		
Baseline CD4+ T counts (cells/μL)						
<200	420	326 (77.62)	Ref.			
200–349	509	415 (81.53)	0.140	1.273 (0.924–1.754)		
≥350	467	374 (80.09)	0.369	1.160 (0.840–1.601)		
Sampling viral load (copies/ml)						
<50	879	698 (79.41)	Ref.			
50–999	82	68 (82.93)	0.450	1.260 (0.693–2.290)		
≥1000	435	349 (80.23)	0.728	1.052 (0.790–1.402)		
Number of non-marital sex partners						
0	866	685 (79.10)	Ref.			
1–4	435	354 (81.38)	0.334	1.155 (0.863–1.546)		
≥5	95	76 (80.00)	0.837	1.057 (0.623–1.793)		
Number of homosexual partners						
0	618	487 (78.80)	Ref.			
1–4	320	264 (82.50)	0.180	1.268 (0.896–1.794)		
≥5	458	364 (79.48)	0.788	1.042 (0.773–1.403)		
Infection of other STD						
No	1069	842 (78.77)	Ref.		Ref.	
Yes	259	212 (81.85)	0.271	1.216 (0.858–1.723)	0.482	1.136 (0.796–1.620)
Unknown	68	61 (89.71)	0.035	2.349 (1.060–5.206)	0.025	2.512 (1.125–5.610)
Drug resistance						
No	1171	923 (78.82)	Ref.		Ref.	
Yes	225	192 (85.34)	0.027	1.563 (1.053–2.321)	0.720	1.084 (0.698–1.682)
HIV-1 subtypes						
CRF01_AE	527	406 (77.04)	Ref.		Ref.	
CRF07_BC	539	441 (81.82)	0.054	1.341 (0.995–1.808)	0.111	1.279 (0.945–1.732)
CRF08_BC	62	49 (79.03)	0.723	1.123 (0.590–2.140)	0.635	1.171 (0.609–2.252)
CRF55_01B	72	60 (83.34)	0.231	1.490 (0.776–2.861)	0.340	1.378 (0.713–2.664)
CRF65_cpx	87	83 (95.40)	<0.001	6.184 (2.222–17.212)	0.003	5.371 (1.793–16.087)
Others	109	76 (69.72)	0.106	0.686 (0.435–1.083)	0.055	0.637 (0.401–1.000)

ART, antiretroviral therapy; STD, sexually transmitted diseases; OR, odds ratio; aOR, adjusted odds ratio; CI, confidence interval; Ref., reference group; CRF, circulating recombinant form.

## Discussion

In this study, we systematically characterized the molecular cluster patterns, spatial hotspots, and cross-regional transmission linkages of HIV-1 in Hainan Province. Our findings revealed widespread cross-regional HIV-1 transmission across the province, with marked geographic variation in clustering rates and intercity transmission links. These spatial differences may be related to population mobility, transportation connectivity, and regional economic activity. By integrating molecular transmission network clustering analysis with city-level spatial analysis, our study provides epidemiological evidence to support geographically targeted HIV prevention and control strategies.

We found that older individuals aged ≥50 years had a higher likelihood of clustering rate in the molecular transmission network. In Hainan Province, HIV infections among the older adults are predominantly acquired through heterosexual contact (Xiaomei et al., 2020). Older adults generally have relatively limited mobility, lower levels of HIV-related knowledge, and weak awareness of self-protection (Zhang et al., 2020). These factors may facilitate transmission within local and relatively stable sexual networks, thereby contributing to higher molecular clustering rates (Zhang et al., 2023). In contrast, individuals with drug resistant had a lower molecular clustering rate, which may be attributable to greater behavioral self-restraint and stricter clinical monitoring after the drug resistance, both of which may reduce onward transmission.

A key finding of this study was the significant interaction between the CRF65_cpx and drug resistance in molecular clustering. In the stratified analysis, after CRF65_cpx was excluded from the group of other subtypes, the interactive effect was eliminated and a significant association was observed. Drug resistant cases of CRF01_AE and CRF07_BC showed relatively low network entry rate. CRF01_AE and CRF07_BC have been the predominant HIV-1 subtypes in Hainan from the early period (Yu et al., 2025). These two subtypes account for a large number of confirmed cases, and are associated with relatively longer treatment duration, which may increase the risk of drug resistance. However, individuals with drug resistance are usually placed under priority follow-up management. Targeted interventions including viral load monitoring surveillance and timely regimen switching, may have substantially reduced onward transmission risks, thereby maintaining low clustering levels for these two subtypes.

At the regional level, Global Moran’s *I* analysis revealed a significant spatial clustering pattern across all HIV-1 subtypes. Locally, LISA analysis identified a high-low clustering pattern for all subtypes in Wuzhishan, suggesting that relatively stable population mobility may facilitate sustained local transmission. A similar high-low pattern was observed for CRF07_BC in Danzhou, where this subtype was primarily identified among unmarried men who have sex with men (MSM). This population is characterized by dense sexual networks and multiple partnerships, which may facilitate the formation of a spatial hotspot. Substantial circulation of the CRF65_cpx occurred mainly in Haikou and was also predominantly associated with MSM. As this subtype has not yet been identified in Lingao, a low-high clustering pattern was observed in this area. These findings suggest that enhanced surveillance in high clustering areas is needed to reduce onward spread into neighboring low clustering regions.

Notably, although Haikou and Sanya had the highest clustering rates and the largest numbers of people living with HIV, they were not identified as hotspot areas in the spatial analysis. This pattern may partly reflect differences in HIV prevention and care capacity. As the major medical center in Hainan Province, Haikou and Sanya may have broader testing coverage, earlier diagnosis, and better access to ART, which could limit sustained local transmission despite high case burdens (McNairy and El-Sadr, 2014). However, given the multiple factor nature of HIV transmission, further investigation incorporating additional behavioral, clinical, mobility, and healthcare-access data are needed to clarify the mechanisms underlying these spatial patterns.

This study found that HIV-1 intercity transmission was common in Hainan, with most transmission links connected to cases in Haikou. The Haikou-Sanya transmission chain was predominant, and active intracity transmission was also observed within both cities. After adjusting for differences in sampling sizes, the Haikou–Sanya pair consistently ranked among the city dyads with the highest molecular linkage intensity. This transmission pattern may be partly driven by socioeconomic connectivity (An et al., 2021). Haikou and Sanya have the highest economic output in Hainan Province, and also account for the largest concentrations of people living with HIV, which may influence HIV transmission dynamics. Therefore, HIV prevention and control efforts should not be limited to individual cities but should also emphasize intercity coordination and cross-regional joint interventions.

We also observed strong CRF07_BC transmission linkages between Haikou and both Tunchang and Dongfang. This may be because CRF07_BC was mainly transmitted among MSM, a population with relatively high levels of HIV-related risk behaviors. These findings highlight the need for targeted health education among MSM, together with active implementation of pre-exposure prophylaxis and ART, to establish an integrated prevention framework for interrupting HIV transmission (Abbas et al., 2013). Continuous surveillance of less active transmission is also needed to prevent further expansion of transmission networks.

Among the individuals within the molecular network, multivariable analysis showed that CRF65_cpx subtype infection was associated with a higher likelihood of cross-regional molecular linkage. In Hainan, this subtype is mainly found among MSM populations, and the higher mobility and social connectivity of MSM networks may contribute to its cross-regional linkage patterns(Jing et al., 2021). Compared with men, women had a lower likelihood of cross-regional molecular linkage, possibly due to differences in mobility and social network characteristics.

The study has several limitations. First, the structure of the molecular transmission network and the spatial patterns of clustering may vary depending on the genetic distance threshold applied, and the inferred HIV-1 transmission network does not necessarily represent direct transmission events. Second, although the sample-size adjusted analysis yielded results generally consistent with the primary analysis, unequal sampling across cities may still have influenced the observed intercity molecular linkage patterns. Future studies with more representative sampling and additional epidemiological data are needed to further validate these findings. Third, in the absence of temporal dynamic analyses, this study failed to identify the migration patterns of HIV-1.

Overall, this study highlights the characteristics of intercity HIV-1 transmission in Hainan, with all subtypes showing spatial clustering patterns, and Haikou and Sanya identified as key areas in intercity transmission. Although no high-high clustering pattern was observed, the potential transition of other clustering patterns into high-high clusters should be considered. Given the prominence of transmission between non-adjacent cities, optimization of health resource allocation is needed, particularly in alignment with regional economic development, alongside strengthened testing services in high-risk areas to improve HIV prevention and control.

## Data Availability

The data analyzed in this study is subject to the following licenses/restrictions: The individual-level original dataset contains sensitive clinical information and blood test results of patients. To protect participant privacy and comply with hospital data management regulations, the raw data cannot be publicly deposited or fully shared online. Access to the de-identified dataset is available to qualified researchers upon reasonable formal application to the corresponding author, with a clear research purpose stated. Requests to access these datasets should be directed to DY, yudee@muhn.edu.cn.

## Footnotes

1
https://www.hiv.lanl.gov/content/index

2
https://comet.lih.lu

3
https://nmdc.cn/hiv
