# Supplementary material for: Molecular transmission network and spatial analysis of HIV/AIDS in Hainan, China: a population-based study
Source: Front Microbiol. 2026 Aug 20;17:1907224. doi: 10.3389/fmicb.2026.1907224 (PMC13539328; doi:10.3389/fmicb.2026.1907224)
Supplement: Supplementary file 1 [file Image_1.pdf]

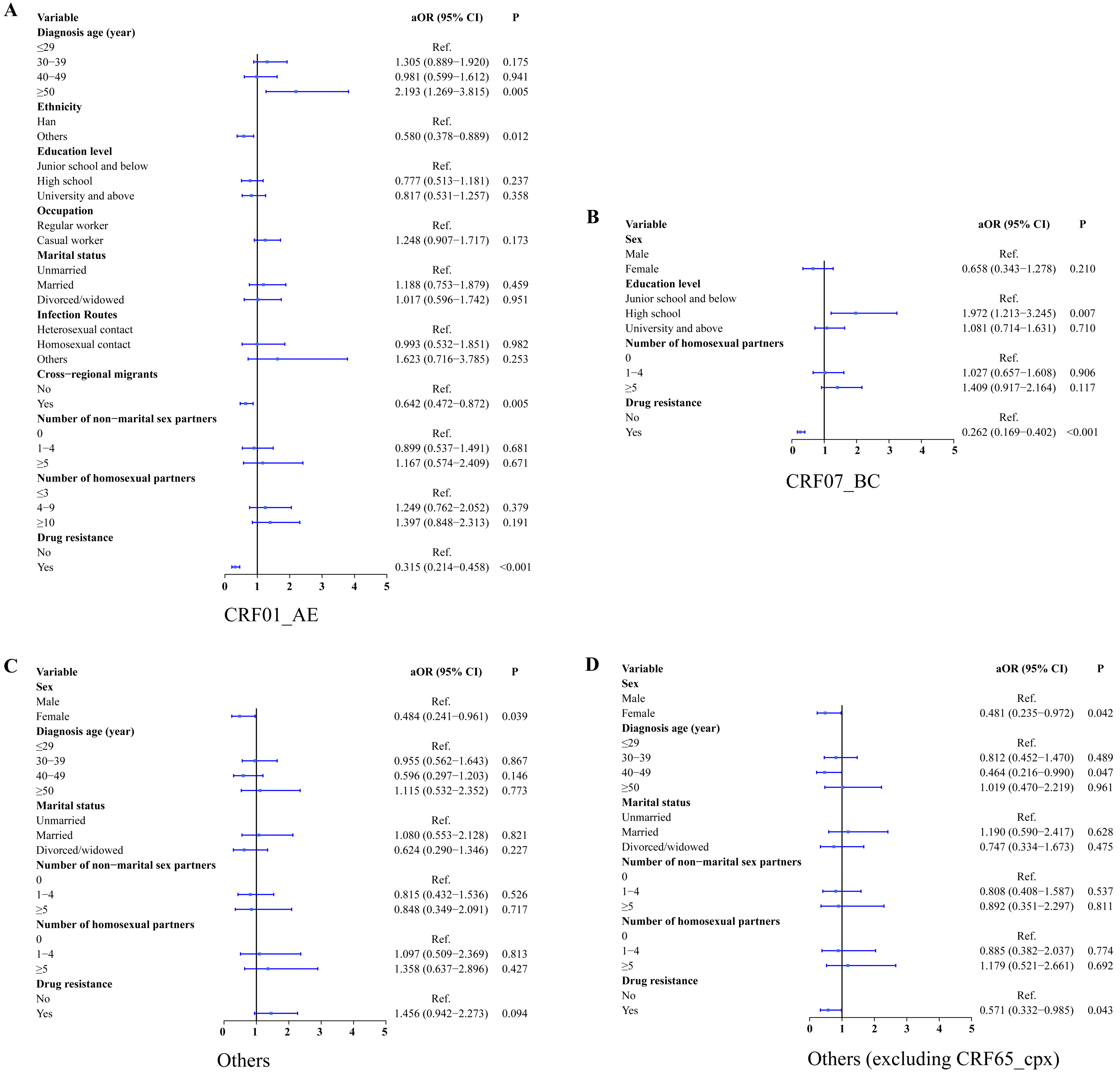

Figure S1. Factors associated with clustering within the HIV 1 transmission network from subtype stratified multivariable logistic regression: only variables significant in univariate analyses (Table S1) were entered into the multivariate models. C: The “Others” group includes CRF08_BC, CRF55_01B, CRF65_cpx and other HIV 1 subtypes. D: CRF65_cpx was excluded from the “Others” group.
