# Supplementary material for: Molecular transmission network and spatial analysis of HIV/AIDS in Hainan, China: a population-based study
Source: Front Microbiol. 2026 Aug 20;17:1907224. doi: 10.3389/fmicb.2026.1907224 (PMC13539328; doi:10.3389/fmicb.2026.1907224)
Supplement: Supplementary file 2 [file Image_2.pdf]

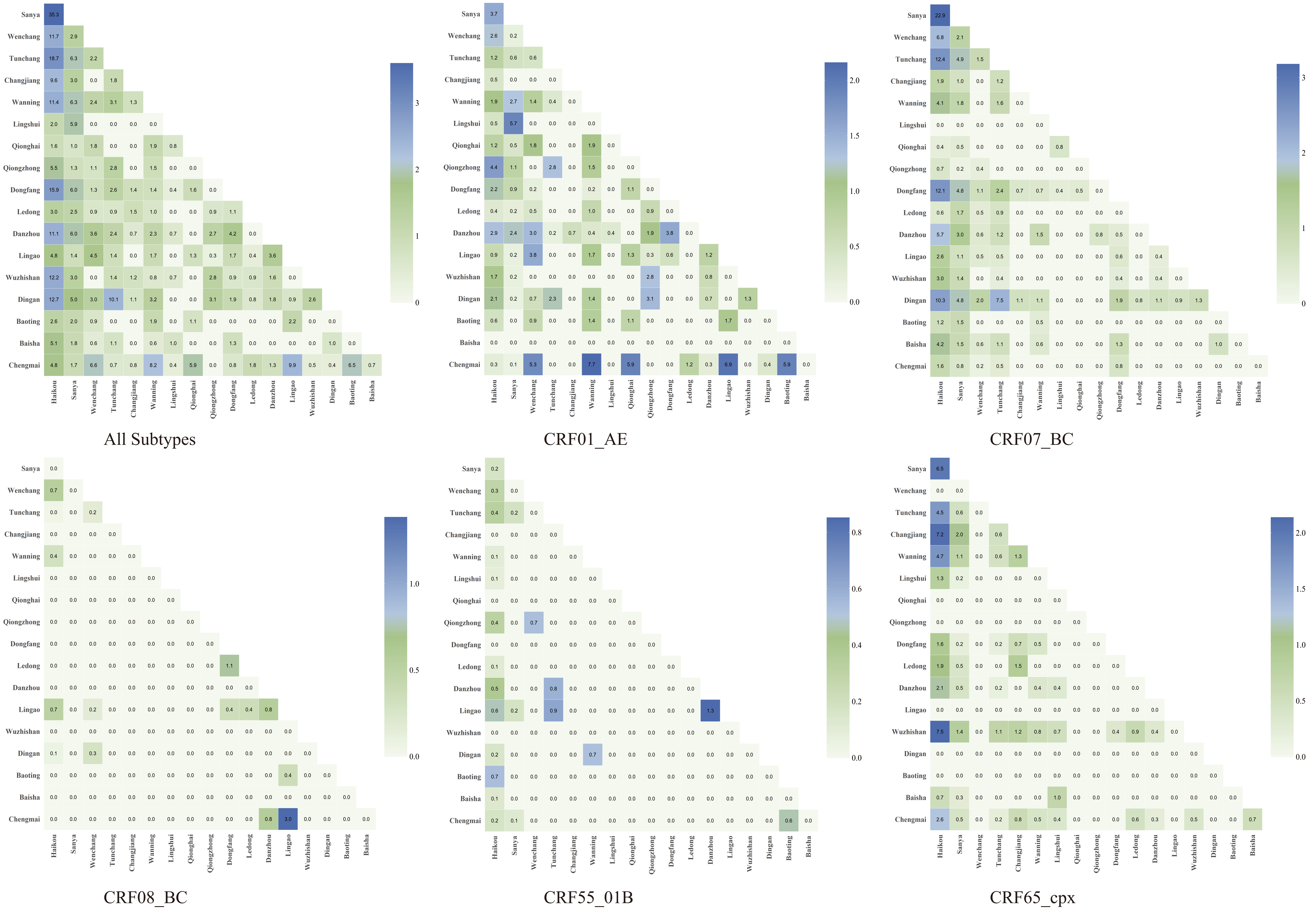

Figure S2. Normalized intercity transmission linkage intensity heatmap. For visualization, the normalized intensity values were multiplied by 100 and a base-10 logarithmic scale was used to enhance colour contrast.
