# Supplementary material for: Molecular transmission network and spatial analysis of HIV/AIDS in Hainan, China: a population-based study
Source: Front Microbiol. 2026 Aug 20;17:1907224. doi: 10.3389/fmicb.2026.1907224 (PMC13539328; doi:10.3389/fmicb.2026.1907224)
Supplement: Supplementary file 3 [file Table_1.docx]

****Table S1.** Interaction analysis between HIV subtype and drug resistance in clustering.**

| Interaction term | Adjusted OR (95% CI) | P |
| --- | --- | --- |
| Subtype*Drug resistance |  | <0.001 |
| CRF01_AE*Drug resistance | Ref |  |
| CRF07_BC*Drug resistance | 0.853(0.487-1.495) | 0.578 |
| CRF08_BC*Drug resistance | 0.515 (0.100-2.094) | 0.378 |
| CRF55_01B*Drug resistance | 2.091 (0.768-5.836) | 0.151 |
| CRF65_cpx*Drug resistance | 81.062 (12.955-685.573) | <0.001 |
| Others*Drug resistance | 1.985 (0.835-4.706) | 0.118 |

****Note:** Adjusted for sex, ethnicity, baseline CD4^+^ T counts (cells/µL), viral load, diagnosis age, and number of homosexual partners.**

****Table S2.** Stratified univariable logistic regression analysis by HIV-1 Subtype.**

| **Variables** | | ****CRF01_AE**** | | ****CRF07_BC**** | | ****Others**** | |
| --- | --- | --- | --- | --- | --- | --- | --- |
|  |  | *****P***** | ****OR (95%CI)**** | *****P***** | ****OR (95%CI)**** | *****P***** | ****OR (95%CI)**** |
| ****Sex**** | | | | | |  |  |
|  | **Male** |  | **Ref.** |  | **Ref.** |  | **Ref.** |
|  | **Female** | **0.296** | **1.243(0.827-1.869)** | **0.047** | **0.552(0.308-0.991)** | **0.001** | **0.389(0.221-0.687)** |
| ****Diagnosis age (year)**** | | | |  | |  |  |
|  | **≤29** |  | **Ref.** |  | **Ref.** |  | **Ref.** |
|  | **30-39** | 0.130 | **1.307(0.924-1.848)** | **0.505** | **1.138(0.778-1.664)** | **0.506** | **0.843(0.510-1.394)** |
|  | **40-49** | 0.694 | **1.087(0.717-1.649)** | **0.978** | **1.007(0.627-1.615)** | **0.002** | **0.418(0.238-0.735)** |
|  | **≥50** | **<0.001** | **3.004(2.034-4.437)** | **0.520** | **1.177(0.716-1.933)** | **0.150** | **0.688(0.413-1.145)** |
| ****Ethnicity**** | | | | | |  |  |
|  | **Han** |  | **Ref.** |  | **Ref.** |  | **Ref.** |
|  | **Others** | 0.007 | **0.581(0.391-0.862)** | **0.342** | **0.799(0.502-1.269)** | **0.457** | **1.241(0.702-2.194)** |
| ****Education level**** | | | | | |  |  |
|  | **Junior school and below** |  | **Ref.** |  | **Ref.** |  | **Ref.** |
|  | **High school** | 0.012 | **0.634(0.444-0.904)** | **0.005** | **1.919(1.213-3.037)** | **0.121** | **1.499(0.899-2.500)** |
|  | **University and above** | 0.001 | **0.592(0.434-0.806)** | **0.223** | **1.253(0.872-1.802)** | **0.139** | **1.378(0.901-2.106)** |
| ****Occupation**** | | | | | |  |  |
|  | **Regular worker** |  | **Ref.** |  | **Ref.** |  | **Ref.** |
|  | **Casual worker** | 0.002 | **1.543(1.169-2.037)** | **0.051** | **0.729(0.530-1.002)** | **0.290** | **1.231(0.838-1.807)** |
| ****Marital status**** | | | | | |  |  |
|  | **Unmarried** |  | **Ref.** |  | **Ref.** |  | **Ref.** |
|  | **Married** | **<0.001** | **1.843(1.342-2.531)** | **0.418** | **1.174(0.796-1.731)** | **0.044** | **0.640(0.415-0.988)** |
|  | **Divorced/widowed** | 0.037 | **1.548(1.026-2.334)** | **0.224** | **0.716(0.419-1.226)** | **0.003** | **0.402(0.222-0.728)** |
| ****Infection Routes**** | | | |  | |  |  |
|  | **Heterosexual contact** |  | **Ref.** |  | **Ref.** |  | **Ref.** |
|  | **Homosexual contact** | 0.009 | **0.691(0.523-0.911)** | **0.139** | **1.282(0.923-1.780)** | **0.001** | **1.942(1.323-2.850)** |
|  | **Others** | 0.492 | **1.261(0.651-2.442)** | **0.142** | **0.402(0.119-1.356)** | **0.717** | **1.374(0.246-7.662)** |
| ****Cross-regional migrants**** | | | | | |  |  |
|  | **No** |  | **Ref.** |  | **Ref.** |  | **Ref.** |
|  | **Yes** | **<0.001** | **0.591(0.446-0.781)** | **0.173** | **1.246(0.908-1.708)** | **0.289** | **0.814(0.555-1.192)** |
| ****Duration of treatment since initial ART(years)**** | | | |  | |  |  |
|  | **<1** |  | **Ref.** |  | **Ref.** |  | **Ref.** |
|  | **1-3** | 0.064 | **0.717(0.505-1.019)** | **0.705** | **0.929(0.635-1.359)** | **0.881** | **0.965(0.608-1.533)** |
|  | **>3** | 0.142 | **0.788(0.573-1.083)** | **0.518** | **1.131(0.779-1.640)** | **0.110** | **0.692(0.441-1.087)** |
| ****Baseline CD4 + T counts (cells/µL)**** | | | |  | |  |  |
|  | **<200** |  | **Ref.** |  | **Ref.** |  | **Ref.** |
|  | **200-349** | 0.717 | **1.060(0.774-1.451)** | **0.430** | **1.179(0.783-1.774)** | **0.141** | **1.409(0.893-2.222)** |
|  | **≥350** | 0.656 | **1.080(0.769-1.517)** | **0.185** | **1.319(0.876-1.988)** | **0.235** | **1.327(0.832-2.117)** |
| ****Sampling viral load(copies/ml)**** | | | |  | |  |  |
|  | **<50** |  | **Ref.** |  | **Ref.** |  | **Ref.** |
|  | **50-999** | 0.121 | **0.629(0.351-1.130)** | **0.321** | **1.427(0.707-2.877)** | **0.615** | **1.222(0.560-2.666)** |
|  | **≥1000** | 0.050 | **1.358(0.999-1.845)** | **0.646** | **1.086(0.763-1.545)** | **0.294** | **1.247(0.826-1.882)** |
| ****Number of non-marital sex partners**** | | | |  | |  |  |
|  | **0** |  | **Ref.** |  | **Ref.** |  | **Ref.** |
|  | **1-4** | 0.608 | **1.078(0.809-1.437)** | **0.620** | **0.916(0.646-1.297)** | **0.021** | **0.619(0.412-0.931)** |
|  | **≥5** | 0.036 | **1.797(1.038-3.110)** | **0.148** | **0.595(0.295-1.202)** | **0.207** | **0.640(0.320-1.280)** |
| ****Number of homosexual partners**** | | | |  | |  |  |
|  | **0** |  | **Ref.** |  | **Ref.** |  | **Ref.** |
|  | **1-4** | 0.003 | **0.601(0.428-0.844)** | **0.559** | **1.124(0.760-1.663)** | **0.038** | **1.669(1.030-2.704)** |
|  | **≥5** | 0.249 | **0.823(0.590-1.147)** | **0.020** | **1.545(1.070-2.230)** | **0.002** | **2.071(1.308-3.278)** |
| ****Infection of other STD**** | | | | | |  |  |
|  | **No** |  | **Ref.** |  | **Ref.** |  | **Ref.** |
|  | **Yes** | 0.434 | **0.865(0.602-1.243)** | **0.078** | **0.710(0.486-1.039)** | **0.265** | **1.311(0.814-2.109)** |
|  | **Unknown** | 0.187 | **0.688(0.395-1.199)** | **0.489** | **0.768(0.364-1.621)** | **0.652** | **0.836(0.382-1.826)** |
| ****Drug resistance**** | | | | | |  |  |
|  | **No** |  | **Ref.** |  | **Ref.** |  | **Ref.** |
|  | **Yes** | **<0.001** | **0.320(0.223-0.459)** | **<0.001** | **0.281(0.185-0.429)** | **0.024** | **1.619(1.067-2.457)** |

**Note:** ART, Antiretroviral therapy; STD, Sexually transmitted diseases; OR, odds ratio; CI, confidence interval; Ref., reference group;
